# Clear cell sarcoma of tendons and aponeuroses of the parapharyngeal space: an unusual localization of a rare tumor (a case report and review of the literature)

**DOI:** 10.11604/pamj.2014.19.147.5364

**Published:** 2014-10-15

**Authors:** Aissa Abdellah, Berhili Soufiane, Bazine Amine, El Majjaoui Sanaa, Elkacemi Hanan, Kharbaoui Ijlal, Latib Rachida, El Khannoussi Basma, Kebdani Tayeb, Benjaafar Noureddine

**Affiliations:** 1 Department of Radiation Oncology, National Institute of Oncology, Ibn Sina University Hospital, Mohamed 5 Souissi University, Rabat, Morocco; 2 Department of Radiology, National Institute of Oncology, Ibn Sina University Hospital, Mohamed 5 Souissi University, Rabat, Morocco; 3 Department of Pathology, National Institute of Oncology, Ibn Sina University Hospital, Mohamed 5 Souissi University, Rabat, Morocco

**Keywords:** Clear cell, sarcoma, rare, parapharyngeal space

## Abstract

The clear cell sarcoma of tendons and aponeuroses (CCSTA) is a rare soft tissue sarcoma in the head and neck region and parapharyngeal space. Over 95% of CCSTAs present in the extremities, with the head and neck region (1.9%) being an unusual site. This study presents an additional case of CCSTA of the head and neck region involving the parapharyngeal space in a 48-year-old men and review of the literature on CCSTA.

## Introduction

Clear cell sarcoma of tendons and aponeuroses, also referred to as malignant melanoma (MM) of soft parts, is a rare malignancy derived from neural crest cells. There is evidence for melanocyte differentiation by immunohistochemical, ultrastructural, and genomic profiling studies [1, 2]. Because of these melanocytic features, its distinction from MM may be difficult. It has a predilection for the distal extremities of young adults, especially the lower limbs, and a high propensity for regional or distant metastases.

## Patient and observation

**Figure 1:**
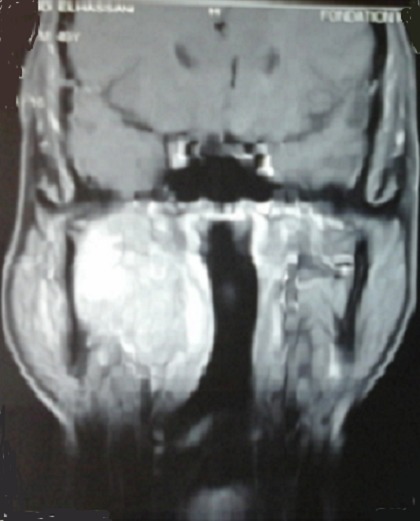
MRI appearance before radiotherapy.

**Figure 2:**
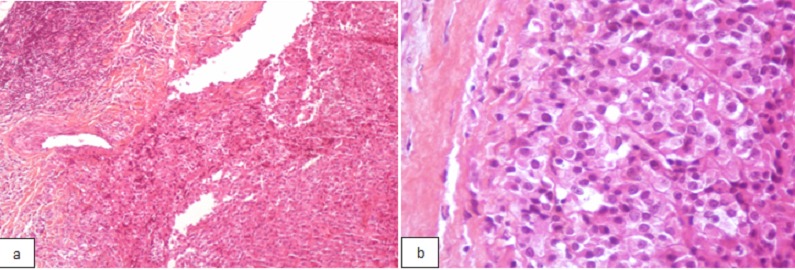
Microphotography showing a compact nests cells with vesicular nuclear chromatin and prominent nucleoli (hematoxylin-eosin Gx40 (a) and GX200 (b)

**Figure 3:**
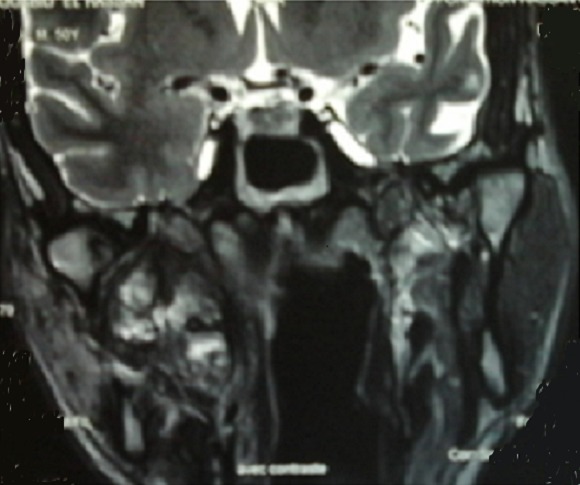
MRI appearance 20 months after radiotherapy

A 48-year-old man presented with a 14-month history of progressive right hearing loss associated with headache and mastication pain. Intraoral examination and rhinoscopy revealed right-sided pharyngeal swelling with normal mucosa on the mass. On intraoral palpation, a relatively well defined and elastic hard mass with no fluctuation was observed. No cervical lymphadenopathy was detected. Computerised tomography (CT) and magnetic resonance imaging (MRI) of the head and neck revealed a well-defined mass measuring approximately 60 mm x 40 mm in its greatest dimension in the right parapharyngeal space (Figure 1). A biopsy through the nasopharynx was performed and the pathologic report was a clear cell sarcoma of tendons and aponeuroses. Histologically, tumors consist of compact nests cells. The cells have highly distinctive features consisting of nuclei with a vesicular nuclear chromatin pattern and prominent basophilic nucleoli. The cytoplasm was eosinophilic weakly clair. The tumor cells were expressing diffusely HMB-45, Melan-A but not expressing PS100, Cytokeratin, EMA, Desmin (Figure 2). No distant metastasis was detected. The patient was evaluated by head and neck surgery, and the tumor was unresectable due to the extensive local invasion. External beam radiation therapy was performed using 6 MV X-rays from a linear accelerator, with daily fraction of 2 Gy, 5 fractions per week and a total dose of 70 Gy was delivered (Figure 3).

**Figure 4:**
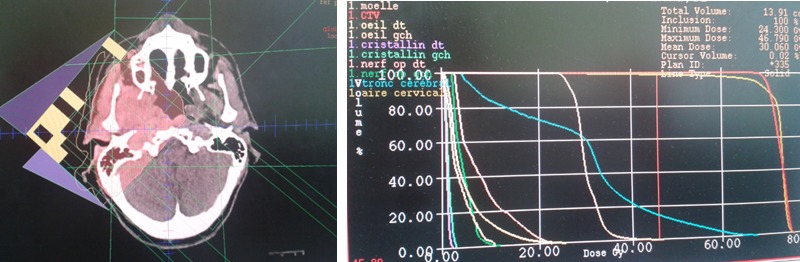
Ballistics used with dose volume histogram

**Figure 5:**
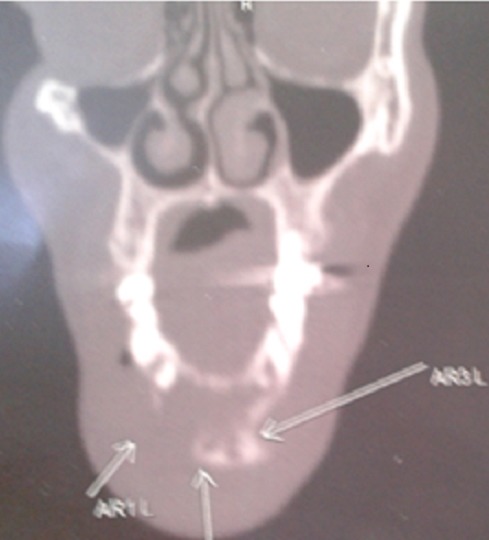
CT appearance of the chin recurrence

During 24 months follow up there is a good clinical response with desperation of headache and pain masticatication. Radiologically, MRI showed significant tumor reduction (Figure 4). Six months later, the patient presented a tumefaction on the chin and CT showed a mass measuring approximately 50mm x 40 mm in diameter (Figure 5). The biopsy showed that is a metastasis of the primary tumor. The lesion is deemed unresectable and the patient received radiotherapy using 6 MV X-rays from a linear accelerator, with daily fraction of 2 Gy, 5 fractions per week and a total dose of 60 Gy.

## Discussion

Described by Enzinger in 1965, the clear cell sarcoma is a rare melanin-producing soft tissue sarcoma [3]. Clear cell sarcoma mainly affects young adults between the ages of 20 and 40 years with a median age of about 30 years. Approximately 40% of cases occur on the foot and ankle with another 30% on the knee, thigh, and hand [4, 5]. The head and neck region, as in our case, are distinctly unusual sites. Only 1.2% of the approximately 500 reported cases of CCS involved the head or neck. [6, 7] because of its close clinic and histologic kinship with malignant melanoma Chung and Enzinger proposed the name malignant melanoma of soft parts [1]. However, despite these similarities, CCS and melanoma should be considered 2 distinct entities. Unlike melanomas, most CCS tumors are characterized by a recurrent chromosomal translocation, t (12; 22), resulting in fusion of the EWS gene on 22q12 with the ATF1 gene on 12q13 [8]. Several fusion transcript types have been described, with a predominance of type 1 fusing exon 8 of EWS with exon 4 of ATF1 and type 2 fusing exon 7 of EWS with exon 5 of ATF1 [9]. The prevalence of t(12; 22) fusion transcripts detected by molecular techniques has been reported in only 3 small series of 10 patients, 12 patients, and 9 patient with CCS [8, 9]. CCS generally evolves slowly and painlessly, and the clinical presentation, which is usually different from MM, is characterized by indolent growth of a soft tissue mass, accompanied by tenderness or pain in 30% to 60% of cases. The best therapy appears to be large excision of the tumor followed by adjuvant radiotherapy [6]. Given the limited number of reported cases, the role of the neck clearance procedure and systemic adjuvant therapy are still uncertain. Five and ten-year survival rates are approximately 47% and 36% respectively [10]. In most cases, the disease pursues a relentlessly progressive course and terminates in death due to wide-spread dissemination. Although rapidly fatal progression may occur, late metastases are quite common after many years of freedom from disease. The most common metastatic sites include the lung, skin, bone, liver and brain [5].

## Conclusion

Clear cell sarcoma of the parapharyngeal space is an extremely rare tumor for which it has been difficult to amass outcome and therapeutic data in a large patient population.
